# Pitfalls in the Diagnosis of Trypanosomiasis in Low Endemic Countries: A Case Report

**DOI:** 10.1371/journal.pntd.0000823

**Published:** 2010-12-21

**Authors:** Leolin Katsidzira, Golden Tafadzwa Fana

**Affiliations:** 1 Division of Medicine, Parirenyatwa Hospital, Harare, Zimbabwe; 2 Department of Medicine, College of Health Sciences, University of Zimbabwe, Harare, Zimbabwe; Institute of Tropical Medicine, Belgium

## Description of Case

A 28-year-old man presented to a referral hospital in Harare, Zimbabwe, with a 6 month history of intermittent fevers and headaches. He worked as a game ranger in the Zambezi Valley. At the onset of his illness, he was treated for malaria with intravenous quinine at a district hospital despite a negative rapid diagnostic test. The symptoms worsened in the 2 months preceding presentation with onset of drenching night sweats and significant weight loss. He had been extensively investigated over this period at a private hospital in Harare, where investigations for HIV infection, diabetes mellitus, and cryptococcal and tuberculous meningitis were negative. A lymph node biopsy had shown reactive changes only. Nevertheless, he was commenced on treatment for tuberculosis. However, he continued to deteriorate, developing progressive generalised rigidity and episodes of confusion. On examination he was very ill, delirious, and febrile with a temperature of 39°C but had no cutaneous lesions and no lymphadenopathy. He had generalised increased tone and the kernig sign was negative. He had brisk reflexes generally and the plantar reflexes were upgoing. However, there were no focal neurological signs and examination of the other systems was unremarkable.

**Figure 1:**
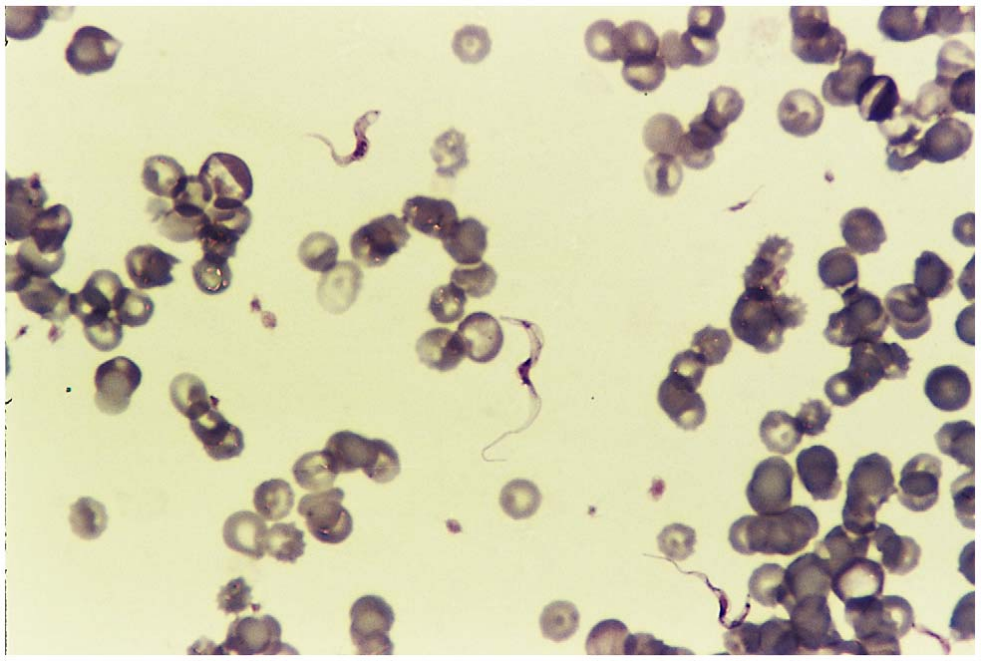
Peripheral blood smear showing *Trypanosome brucei rhodesiense*. (Giemsa×100, oil immersion.)

Human African trypanosomiasis was suspected and confirmed on a thin blood smear (Figure 1). The cerebrospinal fluid (CSF) showed a protein of 4.6 g/L (0.15–0.45 g/L), glucose 4.3 mmol/L (2.8–4.2 mmol/L) and 6 mononuclear cells/mm^3^. No specific search for trypanosomes was performed on the CSF, which was analysed before the diagnosis was made. The blood glucose was 6.1 mmol/L and the full blood count was as follows: white cell count 6.28×10^3^/µL, haemoglobin 7.5 g/dL, and platelets 145×10^3^/µL. The urea and electrolytes were normal. The final diagnosis was stage 2 rhodesiense trypanosomiasis. The specific drugs for this stage (suramin and melarsoprol) were available from the World Health Organization (WHO) emergency country stock. Despite melarsoprol being availed urgently, the patient died, before the initial dose was administered.

Consent: Informed written consent for publication of the case was obtained from the patient's relatives.

## Case Discussion

There is considerable underdiagnosis of human trypanosomiasis caused by *Trypanosoma brucei rhodesiense* in sub-Saharan Africa [1]. This is not easily amenable to quantification in low endemic countries such as Zimbabwe. The majority of the few cases originating from Zimbabwe are diagnosed outside the country, mainly in Britain and South Africa [2], [3]. It is likely that the few cases presenting to local health facilities are getting missed, even though treatment is readily available through WHO initiatives [4]. This is also important for other sub-Saharan African countries with a low disease burden. There are also frequent reports of the disease in visitors to sub-Saharan Africa in general, in whom the diagnosis may also be missed or unduly delayed [5]–[7].

In an epidemic in Uganda, it was estimated that more than a third of cases of rhodesiense trypanosomiasis went undetected and unreported [1]. There were approximately 12 undiagnosed deaths for every reported case. The median time between presentation and diagnosis was 30 days, which implies that lack of access to health care facilities and the health-seeking behaviour of the affected populations were not the only contributing factors. Poor diagnostic facilities, shortage of experienced laboratory personnel, and reduced awareness of the disease among health care workers are probably significant determinants of both underdiagnosis and delayed diagnosis of the disease.

In general, there is a reduced knowledge of neglected tropical diseases among medical practitioners in sub-Saharan Africa [8]. This may be marked in areas where particular conditions are perceived to be uncommon and leads to failure to optimally deploy the available limited diagnostic modalities for maximal benefit. One of the authors involved in this case (LK) received further training in tropical infections, which may have enhanced the final diagnostic process. This potential knowledge gap must be addressed during both initial medical training and through continuing medical education, particularly for health workers in regions at risk for specific conditions.

Specifically, the severe HIV and tuberculosis epidemic in sub-Saharan Africa has also helped push rhodesiense trypanosomiasis from the diagnostic limelight. Numerous neurological conditions with a similar clinical picture occur in HIV infection, and, rightly so, these are the main focus of clinical decision making. Unfortunately, this is frequently accompanied by a failure to consider alternative causes, especially when the HIV tests are negative. This diagnostic blind spot is also seen when malaria is the initial clinical diagnosis, as illustrated in this case. Clinicians in sub-Saharan Africa frequently over-treat malaria, i.e., treating even when sensitive diagnostic tests are negative [9]. The increasing use of these rapid diagnostic tests for malaria is also making routine blood microscopy redundant, inadvertently eliminating the opportunity of making other, incidental diagnoses. The increasing reliance on automated haematological analysis can potentially worsen the problem, and this oversight can occur even in countries with advanced health systems.

## Trypanosomiasis in Zimbabwe

The first case of human trypanosomiasis originating from Zimbabwe, then named Southern Rhodesia, was diagnosed in London in 1911 [10]. The disease has always been uncommon in the country, with only a few sporadic cases reported in the local population annually in the 1960s and 1970s, albeit more regularly than at present [11], [12]. *T. brucei rhodesiense*, which causes an acute, relatively rapidly progressive disease, is the implicated protozoan in Zimbabwe and the rest of southern and eastern Africa. In 1968, it was noted that the epidemiology of the disease in the country had not changed since the initial cases were reported [13]. The disease has always been localised in the Zambezi Valley; between Kariba and the Chewore River, Kanyemba, the Hunyani River area between the Zambezi Escarpment and Mashumbi Pools, and the area north of Mashumbi Pools [12].

The past decade has seen severe political, social, and economic problems in the country, culminating in what is now internationally recognised as the worst hyperinflation in the world in recent times [14], [15]. This resulted in gross under-funding of social and health services, disrupting disease surveillance and control programmes. There has also been an accelerated skills flight adversely affecting all sectors of the health and ancillary systems. Not surprisingly, there has been a re-emergence of previously well-controlled diseases such as measles and cholera [16], [17]. It is crucial to ensure that this case is not just the tip of the iceberg, as there is no active surveillance for trypanosomiasis in the country.

## The Presenting Case

The role of history in clinical medicine cannot be overemphasized. In this case, the patient lived in the right geographical region and his occupation placed him at heightened risk of contracting trypanosomiasis. Consideration of this should have triggered appropriate investigations much earlier in the disease course. Generally with tropical infections, exposure to particular geographical areas is crucial in guiding initial investigations and treatment. This maxim is equally relevant in both developing and developed countries and can be lifesaving. Finally, this case demonstrates the continued relevance of microscopy as newer, faster diagnostic methods for various diseases are being developed and introduced. This vital skill should not be left to wane, particularly in frontline hospitals in resource-limited settings.

**Learning Points**

- History of relevant exposure is the cornerstone of the diagnosis of most tropical infections.
- Microscopy is a vital skill that should be preserved as new diagnostic methods for malaria and tuberculosis emerge.
- Political and social instability provides the right milieu for the reemergence of previously controlled diseases.
- Clinicians in areas where HIV, tuberculosis, and malaria are common must remain open-minded regarding other, less common but severe and potentially fatal treatable conditions.
